# Unraveling Pediatric Forearm Trauma: A Case Study on Diagnostic and Management Challenges

**DOI:** 10.7759/cureus.84727

**Published:** 2025-05-24

**Authors:** Robin Dezes, Paul Boulos, Simon Kalisz, Joe Kadou

**Affiliations:** 1 Emergency Medicine, Chirec Delta Hospital, Brussels, BEL

**Keywords:** elbow trauma, pediatric emergency care, pediatric forearm fracture, trauma pediatric, upper extremity trauma

## Abstract

Forearm trauma in children is a frequent reason for emergency department visits. An often incomplete medical history can lead to difficulties in understanding the traumatic mechanism. This case study highlights the complexity of managing pediatric forearm injuries, particularly due to the potential presence of associated lesions, which must be thoroughly investigated through clinical and radiological examination. In this context, this case serves as a reminder of the different types of forearm and elbow fractures, the potentially associated injuries that should be systematically sought, and the clinical and radiological tools available to the emergency physician.

## Introduction

Traumatology remains a major indication for consultation in pediatric emergency care settings and represents one of the primary causes of morbidity and mortality in children [1]. In 2019, trauma accounted for 47% of emergency visits in patients under the age of 15 in France [2]. The majority of unintentional injuries in children are associated with falls [3,4]. Among these traumas, forearm fractures are the most frequent, whereas elbow fractures represent only 16% of all pediatric fractures [5]. The challenge in managing pediatric forearm trauma in the emergency setting is to avoid missing associated fractures or dislocations in the same limb. A comprehensive clinical examination, specifically the examination for deformity or palpation-induced pain, is essential [6]. Subsequently, appropriate imaging is essential to establish an accurate diagnosis and to provide appropriate treatment. This step involves obtaining radiographs or a computed tomography (CT) scan, especially for the pre-operative assessment of complex fractures [7].

## Case presentation

This is the case of a 14-year-old male with no significant medical or surgical history who presented to the emergency department with left wrist and elbow pain following a fall on a trampoline. The exact characteristics of the fall were not clearly described by the patient or his parents at the time of the history-taking.

The initial clinical examination revealed a visible dorsal concavity deformity of the left forearm, with pain on palpation of the forearm, elbow, and wrist. The pain was elicited with minimal movement. Additionally, hypoesthesia was noted in the fourth and fifth digits of the left hand. No vascular deficit was observed, distal coloration was preserved, and both ulnar and radial pulses were palpable.

**Figure 1:**
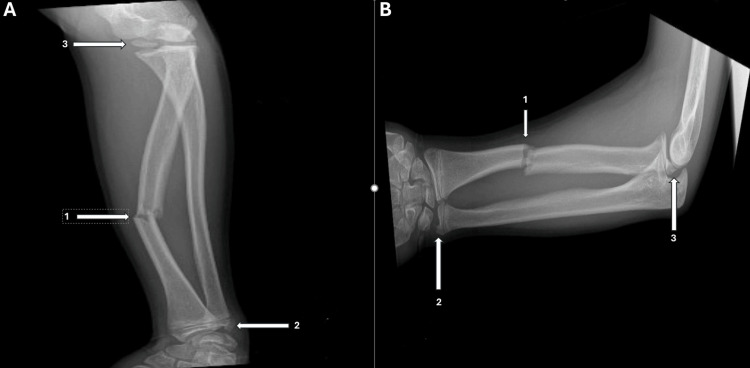
X-ray of the forearm, elbow, and wrist (anteroposterior and lateral view) A: Anteroposterior X-ray showing a fracture with dorsal angulation of the radial diaphysis (arrow 1) associated with avulsion of the ulnar styloid (arrow 2) and avulsion dislocation of the epiphyseal nucleus of the epicondyle (arrow 3). B: Lateral X-ray showing a fracture with dorsal angulation of the radial diaphysis (arrow 1) associated with avulsion of the ulnar styloid (arrow 2) and avulsion dislocation of the epiphyseal nucleus of the epicondyle (arrow 3).

Radiographs of the left forearm and elbow (Figure 1) revealed a displaced diaphyseal radial shaft fracture associated with an avulsion-dislocation of the ulnar styloid, consistent with a Galeazzi fracture. Additionally, an intra-articular bone fragment was visible on the medial aspect of the elbow joint.

**Figure 2:**
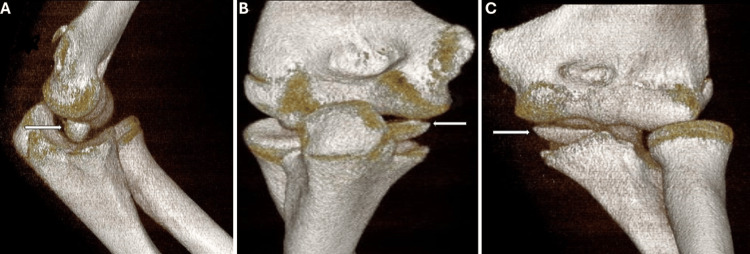
3D reconstruction on CT of the left elbow (lateral, posterior, and anterior view) A: Lateral view showing a fracture-avulsion of the epiphyseal nucleus of the epicondyle with dislocation of the fragment at the intra-articular level between the medial humeral condyle and the ulna (arrow). B: Posterior view showing a fracture-avulsion of the epiphyseal nucleus of the epicondyle with dislocation of the fragment at the intra-articular level between the medial humeral condyle and the ulna (arrow). C: Anteroposterior view showing a fracture-avulsion of the epiphyseal nucleus of the epicondyle with dislocation of the fragment at the intra-articular level between the medial humeral condyle and the ulna (arrow).

To further evaluate the extent of the injury, a CT scan of the left elbow (Figure 2) was performed, which revealed an avulsion fracture of the epiphyseal nucleus of the medial epicondyle, as well as an intra-articular dislocation of the fragment between the medial humeral condyle and the ulna.

The mechanism of the traumatism was likely biphasic: a direct injury caused the Galeazzi fracture, followed by a landing with the elbow in hyperextension and valgus position, leading to the fracture and intra-articular avulsion of the epiphyseal nucleus of the medial epicondyle.

The diagnosis was a left Galeazzi fracture associated with a stage III Watson-Jones elbow fracture-dislocation and ulnar nerve hypo-paresthesia.

Due to the urgent surgical indication, the patient underwent same-day surgery, including open reduction and internal fixation (ORIF) of the radius and the medial epicondyle, as well as ulnar nerve decompression. Follow-up clinical and radiological monitoring was arranged.

**Figure 3:**
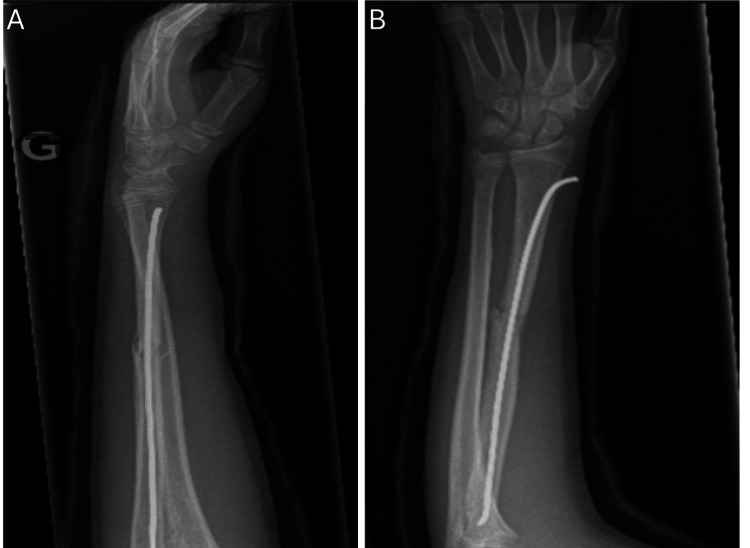
Four-week radiograph showing a mid-diaphyseal radial fracture undergoing consolidation, with pin osteosynthesis and anatomical realignment A: Lateral view B: Anteroposterior view

Two weeks post-operative, the ulnar nerve territory showed a slow, gradual recovery. At four weeks, X-rays showed signs of radial fracture healing with preserved alignment (Figure 3). However, ulnar neuropathy persisted.

A second surgical intervention was then performed to reexplore the ulnar nerve. Intraoperative findings revealed no discontinuity of the nerve but inflammatory tissue around the arcade of Struthers and the arcuate ligament. Debridement of this tissue and ulnar nerve neurolysis were performed.

Three months later, the patient showed good ulnar nerve recovery, and implant removal was planned. Five months post-trauma, neurological recovery was complete, and bone healing was satisfactory.

The patient was scheduled for a clinical and radiological follow-up at 12 months post-injury.

## Discussion

There are various types of elbow fractures [8], such as supracondylar fractures (60%), medial epicondyle fractures (10%), lateral condyle fractures (10%), radial neck fractures (10%), olecranon fractures (5%), medial condyle fractures (1-2%), capitellum fractures (1%), T-shaped intercondylar fractures (1%), and elbow dislocations (3%).

According to the 2007 recommendations from the Société Française de Médecine d’Urgence (French Society of Emergency Medicine) on the management of elbow trauma in the emergency department [6], it is necessary to perform a thorough clinical examination, including assessment for deformities and pain on palpation of the medial and lateral epicondyles and olecranon apophysis. Palpation of the humeral head and ligamentous structures, particularly the medial collateral ligament, is essential in suspected dislocations. Examination of the distal radioulnar joint is also necessary.

Wounds that could pose an infectious risk must be checked. A thorough neurovascular examination is also critical. It is necessary to carefully assess the vascular and nerve structures of the limb by checking the distal, radial, and ulnar pulses, analyzing the time of skin recoloration at the extremities, as well as the humeral pulse on the anterior side of the elbow. The sensitivity and motor function of all fingers should be tested.

According to the 2015 Belgian Public Health recommendations regarding radiological assessment of pediatric trauma [7], standard anteroposterior and lateral radiographs should be performed. CT scans can be used for complex fracture characterization. Ultrasound, except for the characterization of the articular cartilage at the humeral condyle in Salter III and IV fractures, does not have a routine place in pediatric trauma to this day.

Approximately 25% of elbow injuries associated with forearm fractures are believed to be missed during the initial radiographic evaluation [8].

Medial epicondyle fractures typically result from a fall on an outstretched hand with the elbow in a valgus position. The medial epicondyle is displaced due to traction by the epitrochlear muscles. According to the Watson-Jones classification, there are four grades of medial epicondyle fractures (Table 1), with our patient presenting a grade III fracture. Management depends on the grade [9]: grade I involves a brachioantebrachial splint cast in a pronation position to relax the epitrochlear muscles with a Dujarrier splint; grades II and III require ORIF. Percutaneous pinning is discouraged due to ulnar nerve injury risk. Grade IV requires ORIF and valgus stability testing. Immobilization typically lasts five to six weeks.

**Table 1:** Watson-Jones Classification Credit: Table created by the authors

Watson Jones grades	Description
I	Fragment not displaced or <5 mm
II	Displacement >5 mm
III	Intra-articular entrapment of the fragment
IV	Associated elbow dislocation

Medial epicondyle fractures are frequently associated with elbow dislocations [10], which was the case with our patient. It is important to check for a possible fracture of the medial epicondyle in cases of elbow dislocation in children. Furthermore, as mentioned in this clinical case, a fractured epitrochlear nucleus can become incarcerated within the joint, making reduction difficult [11]. Therefore, it should always be investigated on radiographs in children over six years old.

Associated injuries in the elbow or wrist should be systematically investigated in forearm fractures. Indeed, according to Soderlund et al. [12], Monteggia fractures, which involve a fracture of the ulnar shaft and an anterior dislocation of the radial head, account for 1-2% of forearm fractures. Similarly, Galeazzi fractures, which represent 3% of forearm fractures in children, involve a fracture of the distal third of the radius and a dislocation of the distal radioulnar joint [13]. Taking this into account, it is necessary to perform radiographs of the elbow and wrist in cases of forearm fractures in children, ideally in the same view [14].

Ulnar nerve injury can occur with elbow trauma, particularly in medial epicondyle fractures [15], presenting as paresthesia in the fourth and fifth digits, sensory deficits, and weakness in the interosseous and hypothenar muscles. Clinical signs include Wartenberg’s sign, Jeanne’s sign, Froment’s sign, and Bouvier’s maneuver.

To this date, to the best of our knowledge, there is no similar case of Galeazzi fractures combined with medial epicondyle fracture-dislocation that has been reported in the literature.

## Conclusions

This case involving a 14-year-old patient emphasizes the importance of thorough clinical examination in pediatric trauma, especially when the history is limited. An obvious forearm fracture may be associated with elbow or wrist injuries. Elbow dislocation in children should raise suspicion for concurrent medial epicondyle fractures. Therefore, systematic assessment of potential vascular and nerve lesions, especially in elbow trauma, is essential to ensure rapid orthopedic management and a favorable prognosis. When diagnostic doubts persist, complementary imaging with a CT scan should be discussed.
